# Are College Students Interested in Family Health History Education? A Large Needs Assessment Survey Study

**DOI:** 10.3390/ijerph20032596

**Published:** 2023-01-31

**Authors:** Ming Li, Oi-Man Kwok, Ping Ma, Tung-Sung Tseng, Lei-Shih Chen

**Affiliations:** 1 Department of Health Sciences, Towson University, Towson, MD 21252, USA; 2 Department of Educational Psychology, Texas A&M University, College Station, TX 77843, USA; 3 Department of Health Behavior, School of Public Health, Texas A&M University, College Station, TX 77843, USA; 4 Behavioral and Community Health Sciences Program, School of Public Health, Louisiana State University Health Sciences Center, New Orleans, LA 70112, USA

**Keywords:** family health history, college students, education, needs assessment

## Abstract

Family health history (FHH) is an essential foundation for personalized disease prevention. As the incidence of early-onset chronic diseases is increasing among college students, it is important to provide them with the education required to learn about their FHH. This study aimed to assess college students’ interest in receiving FHH education, preferred topics, and desired learning methods. We invited college students to complete an online survey from a large research-intensive university. A total of 2276 college students completed the survey. Nearly half of the participants self-identified as non-Hispanic white (45.5%). Slightly more than half of the sample (53.9%) were not interested in receiving FHH education mainly due to low prioritization. Among those who expressed interest in obtaining FHH education, the three most desired learning topics were the ability to interpret FHH information (76.1%), the application of FHH in disease prevention (72.0%), and FHH information collection strategies (63.6%). Computer-based learning (51.1%) was the most preferred educational method. Moreover, females, older individuals, those who have FHH in first-degree relatives, and participants who were members of racial and ethnic groups showed greater interests in receiving FHH education (*p*s < 0.05). Strategies to promote college students’ awareness, collection, and use of FHH are needed.

## 1. Introduction

In the era of precision medicine, family health history (FHH) is the most cost-effective and powerful genomic tool [1], and the foundation of personalized disease prevention [2]. As FHH reflects the interactions of genes, behaviors, and environment, FHH is an important risk factor for many diseases [1,3]. Individuals should first collect their FHH from at least three degrees of relatives. Based on the FHH information, healthcare providers and public health professionals can provide personalized medical and lifestyle recommendations to prevent disease. In addition, a number of studies suggested that an accurate and comprehensive FHH can motivate individuals to adopt healthy behaviors, such as exercise, healthy eating, and undergoing regular medical screenings [4,5,6,7,8,9,10,11,12]. Existing FHH-based health interventions targeting the young adult population [4,7,13,14,15] also demonstrated that FHH interventions can successfully increase participants’ knowledge towards FHH, increase their understanding towards personal disease susceptibility, and motivate the adoption of healthy behaviors.

As the incidence and prevalence of early-onset chronic diseases (e.g., cancer, diabetes, and obesity) are increasing among college students, it is important to educate them on how to gather and learn from their FHH, and modify their lifestyle based on their FHH for three main reasons. First, many chronic diseases or conditions can be prevented by adopting and maintaining healthy behaviors. Yet, because college students usually perceive themselves as being healthy and have low awareness of their potential health risks and related negative health consequences, they face a significant barrier in the use of FHH [16]. Second, approximately half of the college students in the United States (U.S.) do not meet the Physical Activity Guidelines for Americans’ recommended levels of physical activity (at least 150 min moderate-intensity or 75 min vigorous-intensity aerobic physical activity per week), and also do not meet the recommended dietary guidelines set forth in the Dietary Guidelines for Americans [17,18,19]. Having accurate FHH information may enable college students to understand their disease risk, tailor preventive strategies, identify appropriate preventive behaviors, and receive personalization in healthcare [20]. Third, as most college students live away from home for the first time, they have begun to have autonomy in, and responsibility for, dietary choices, weight management, and physical activity [17,21]. It is critical to educate these young adults about their FHH, and help them to establish healthy habits that will continue in later life [21,22].

To effectively educate college students, information must be first collected to understand their interest in receiving FHH education, and their preferred FHH informational topics and desired learning methods. This is particularly important as today’s college students face many competing demands on their time including academic and social activities, and multiple other concurrent commitments [23]. Such a needs assessment will assist in the development of an effective FHH-based educational program for college students in the future. The purpose of this, to the best of our knowledge, first-of-its-kind, study is to seek answers to the following questions: (1) Do college students perceive the needs of receiving FHH education, (2) what FHH informational topics are college students most interested to learn, and (3) what is their preferred learning strategy for FHH education?

## 2. Materials and Methods

### 2.1. Survey Instrument

This study is a part of a research project designed to assess college student FHH collection behaviors and educational needs [24]. A web-based survey was developed based on the literature to examine factors related to FHH collection behavior and interest in FHH education among college students [16,25,26,27,28,29,30,31,32,33]. The draft of the survey was then reviewed by a panel of experts to ensure content validity. This panel included experts from statistics, health education, health behavior, health communication, and college health. The revised survey was tested through cognitive and retrospective interviews with nine and eight college students, respectively. Next, the survey was pilot-tested with 63 college students recruited from two undergraduate courses. The survey was refined by revising several questions for clarification and removing confusing items according to feedback received from participants in the pilot test.

### 2.2. Data Collection

The survey data were collected through Qualtrics, a web-based survey platform (http://www.qualtrics.com, accessed from 15 October 2018 to 20 November 2018). Responses were anonymous and participation was voluntary. Eligibility participation criteria included: (1) undergraduate and graduate students who were young adults between the ages of 18 and 35 years, and (2) students who were registered on either of two campuses of a large, public, research-intensive university in the southern region of the U.S. A total of 55,346 students received an initial recruitment email and three follow-up reminder emails with the survey link through the university bulk email service. When participants completed the survey, they received a link to a separate survey that could not be traced back to the initial survey to enter their names and emails for incentives. The incentives were distributed through a random drawing for forty 50 USD electronic gift cards. Additionally, the first 100 participants who completed the survey each received a 5 USD electronic gift card. A total of 2809 students participated in the survey yielding a response rate of 5.08%. Participants who missed the entire needs assessment items and/or those whose age was below 18 or over 35 were excluded. Thus, the final sample consisted of 2276 college students. The study protocol was approved by Texas A&M University’s Institutional Review Board prior to the collection of any data.

### 2.3. Measures

Interest in FHH education. Adopted from a past study [30], interest in FHH education was assessed by asking “how interested would you be in participating in an FHH educational program in the future that will assist you in collecting your FHH, understanding your risk level, and obtaining personalized disease prevention recommendations based on this risk?” [1 = “not at all interested”; 5 = “extremely interested”]. To assist participants who were not familiar with the topic of FHH to understand this question, a brief explanation that summarized the major use and application of FHH in disease prevention was added to the measurement. Only the participants who reported being “interested,” “very interested,” or “extremely interested” were navigated on to the questions regarding desired FHH informational topics and preferred educational strategies. 

Desired topics in FHH education. Participants’ desired FHH education topics were measured by asking them to choose from four themes that were adopted from the literature [32]. These four themes included: “What is FHH?”, “How can I collect my FHH”, “Why is FHH important for my health?”, and “How can I use my FHH to improve my health”. We added a fifth option, “How can I interpret my FHH results?” based on participant feedback we received from the pilot test. We also provided an open-ended “other” option for participants to describe their preferred topics. Participants could choose more than one theme if desired. 

Preferred FHH educational strategies. We asked participants to choose their preferred FHH educational strategies from a list of eight teaching strategies (i.e., traditional lectures, discussions, simulated games, computer technologies, written materials, audiovisual sources, demonstration, role playing, and others). These strategies were selected based on a systematic review of effective health education teaching strategies and methods of delivery [34]. Participants could choose more than one option if desired. 

FHH of major diseases in three-degree relatives. All participants were asked to report their FHH of 15 major diseases (e.g., cancer, dementia, diabetes, and heart diseases) listed by the U.S. Surgeon General’s “My Family Health Portrait” [35] in three degrees of relatives in three separated matrixes (e.g., “Please select if any of your first-degree relatives (FDR)/second-degree relatives (SDR)/third-degree relatives (TDR) has experienced or is expiring any of the following conditions” [no, my FDR/SDR/TDR has not experienced this condition; yes, my FDR/SDR/TDR has experienced or is expiring this condition; I do not know/not sure]). If participants reported any first-degree/second-degree/third-degree relatives with any of the above 15 major diseases, they were coded as having an FHH of major diseases in first-degree/second-degree/third-degree relatives, respectively. We also provided the definition of first-degree/second-degree/third-degree relatives before each matrix to help participants answer the questions. 

Lifestyle. The survey included validated measurements regarding fruit consumption (0 cup, 0.5 cup or less, 0.5 to 1 cup, 1 to 1.5 cups, 1.5 to 2 cups, 2 to 2.5 cups, 3 or more cups each day); vegetable consumption (0 cups, 0.5 cup or less, 0.5 to 1 cup, 1 to 1.5 cups, 1.5 to 2 cups, 2 to 2.5 cups, 3 or more cups each day); weekly red meat intake (in ounces); weekly processed meat intake (in ounces); alcohol consumption (no or yes); current smoking status (no, yes, or smoked before but have now quit); and physical activity (frequency of exercise per week multiplied by the average duration for each time) [12,31,36,37]. 

Genetic/genetic-related course(s) at college. We asked participants if they had taken a course in genetics or genomics at college (no or yes) and had ever enrolled in a course containing genetics/genomics-related information in college (no or yes) [38]. 

Sociodemographic characteristics. The survey measured participants’ age, gender, birthplace, race/ethnicity, and marital status.

### 2.4. Statistical Analysis

Descriptive statistical analysis was conducted to measure the frequencies of participant responses to each survey item. Bivariate correlations were conducted to examine the relationships between FHH education interests, continuous variables (i.e., age, fruits and vegetable consumption, intake of red and processed meat, and physical activity level), and categorical variables (i.e., gender; birthplace; race/ethnicity; religions; marital status; alcohol consumption; current smoking status; FHH of major diseases in first-degree relatives, second-degree relatives, and third-degree relatives; whether or not having taken genetics/genomics courses in college; and whether or not they have a course containing genetics/genomics-related information in college). Only those variables found to be statistically significant in bivariate analysis (i.e., age, gender, birthplace, ethnicity, marital status, fruit consumption, red meat intake, FHH of major diseases in first-degree relatives, whether or not having taken a genetics/genomics course in the college, and whether or not having taken a course containing genetics/genomics-related information) were then included in the multiple linear regression analysis. All statistical programming was conducted using STATA Version 15.0 with *p* < 0.05 as the significance threshold.

## 3. Results

### 3.1. Sample Characteristics

Table 1 summarizes the sociodemographic characteristics of the sample. Respondents are predominantly female (66.0%), Christian (64.3%), born in the U.S. (78.9%), and have a mean age of 21.0 years (SD = 3.4, range: 18–35). Nearly half of participants self-identify as non-Hispanic white (45.5%). Fewer than one in five of the college students in our sample (15.6%) report having taken a genetics or genomics course in college, while more than twice as many participants (33.7%) report having taken a course containing genetics or genomics information.

### 3.2. Interest in Receiving FHH Education

**Figure 1:**
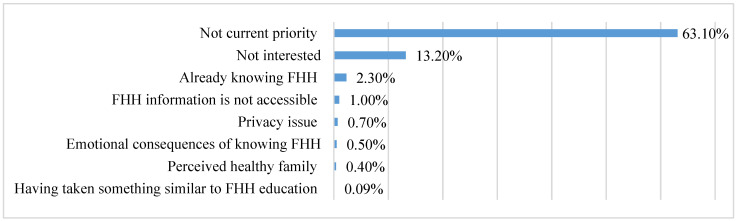
Reasons for not being interested in receiving FHH education (*n* = 1226). FHH: family health history.

We asked participating college students if they would be interested in receiving FHH education. Fewer than half of participants (46.13%) reported that they were “interested” (25.26%), “very interested” (13.44%), or “extremely interested” (7.43%) in having access to such education. The majority of the survey respondents stated that they were not interested in FHH education (53.87%). Those respondents who were not interested in FHH education were asked to provide reasons why they were not interested. As summarized in Figure 1, those reasons, listed by the frequency from highest to lowest include: (1) not a priority topic at present (63.10%); (2) lack of interest in the topic of FHH (13.20%); (3) already knowing their own FHH (2.30%); (4) lack of ability to access FHH information from family members (1.00%); (5) privacy concerns (0.70%); (6) the emotional consequences of knowing their FHH (0.50%); (7) perception that their family was healthy (0.40%); and (8) having already participated in previous FHH-related education (0.09%). 

As shown in Table 2, the multiple linear regression reveals that older and female students and those with an FHH of major diseases in first-degree relatives are more interested in receiving FHH education (β = 0.021, *p* < 0.01; β = 0.206, *p* < 0.001; β = 0.185, *p* < 0.05, respectively). Students who self-identify as being of diverse racial and ethnic groups also have more interest in FHH education than did non-Hispanic white students (β = 0.105, *p* < 0.05).

### 3.3. Desired FHH Educational Topics

**Figure 2:**
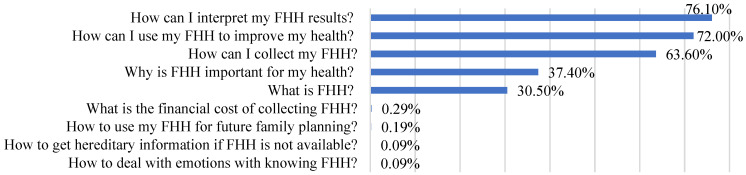
Participants’ preferred FHH educational topics (*n* = 1050). FHH: family health history.

We asked participants who expressed interest in receiving FHH education the FHH information topics that were of greatest interest to them. As illustrated in Figure 2, the five most frequently reported FHH informational topics ware: (1) How can I interpret my FHH results (76.1%)? (2) How can I use my FHH to improve my health (72.0%)? (3) How can I collect my FHH (63.6%)? (4) Why is FHH important for my health (37.4%)? (5) What is FHH (30.5%)? Other topics mentioned by some participants include: the financial costs of collecting FHH (0.29%), how to use FHH in family planning (0.19%), how to obtain genetic information if FHH is not available (0.09%), and how to handle the emotions associated with knowing the results of a FHH (0.09%).

### 3.4. Preferred FHH Educational Strategies

For those participants who expressed interest in receiving education about FHH (*n* = 1050), we asked the type or methods of education they preferred. Computer technology (e.g., computer-based education or computer-assisted learning) is reported by participants as their most preferred strategy (51.1%). Other desired educational methods include demonstrations (44.2%), traditional lectures (43.3%), discussions (42.3%), written materials (41.5%), simulated games (36.8%), audiovisual sources (36.1%), and role playing (14.8%). Majority of the participants (81.0%) choose more than one educational strategy. The most popular combinations include “discussion” and “demonstration” (23.2%), “simulated games” and “computer technology” (23.1%), “computer technology” and “demonstration” (22.4%), “traditional lectures” and “written materials” (21.6%), “computer technology” and “written material” (21.5%), and “simulated games” and “demonstration” (20.5%).

## 4. Discussion

Results from this study make a significant contribution to the literature by increasing our understanding of the FHH-related educational needs of young adults. Educating young adults about FHH is fundamental to the practice of precision medicine. More than half of the participants in our study, however, report no interest in receiving education about FHH. The main reason reported for this lack of interest is that learning about FHH is not a current priority in their lives. This finding is in line with a previous study that reported that college students, a young and relatively healthy population, tend to be unrealistically optimistic about their health risks [39,40]. As a consequence, this optimism bias undermines their motivation to, and interest in adopting the precautions necessary to reducing the risks of a wide range of diseases [40,41]. According to the transtheoretical model, health messages and programs should be uniquely designed and tailored to the targeted population according to their stage of change (i.e., precontemplation, contemplation, preparation, action, and maintenance) [42]. To increase the success of FHH educational efforts on college campuses, FHH education may be customized for young adults based on their different motivational and interest levels in the topic of FHH. For example, young adults with a high motivation level should be offered education focused on helping them prepare to collect FHH and using it in health promotion. For young adults who are not yet ready to pursue FHH education, it is critical to deliver a message describing the importance and relevance of FHH to health. According to our findings, lack of priority of FHH is the major reason why college students are not interested in FHH education. Thus, future FHH education for college students may consider incorporating it with the existing curriculum (such as a health introduction course) or other health interventions (such as nutrition or physical excise education). 

Our findings show that female and relatively older participants are more interested in FHH education. These results are consistent with previous works in the literature that report that females and older people usually play active roles in collecting and communicating FHH with family members [16,43,44,45]. A survey study examined young adults (aged between 18–29) and found that compared with younger adults, older adults tended to have more positive attitudes regarding the power of FHH on personal health outcomes, and are more likely to report an interest in using FHH collection tools [45]. Furthermore, among limited FHH research focused on young adult population, the majority of their participants were female [4,7,14,15,45]. This may explain why these groups in our study might be more motivated to seek FHH education. Therefore, in future research, it is important to recruit more male participants to understand their perspectives and needs regarding FHH education. Moreover, participating college students who reported an FHH of major disease in their first-degree relations were more likely to be interested in obtaining FHH education. This finding is consistent with previous results from a community-based survey of adult participants that reported a significant association between participants’ awareness of their own FHH and their engagement with FHH-related health education [46].

Interestingly, our data indicate that survey respondents who self-identify as members of diverse racial and ethnic groups are more motivated to seek FHH education than non-Hispanic white respondents. This high level of interest could be related to the fact that college students who are members of diverse racial and ethnic communities have a stronger intention to collect FHH than non-Hispanic white participants [24]. Past research in the literature also suggested that members of diverse racial and ethnic groups possessed limited knowledge of FHH and had low levels of FHH collection [47,48]. Those findings may possibly explain why participants from diverse racial and ethnic groups are more interested in FHH education. Future studies should explore the underlying reasons that explain why members of diverse racial and ethnic groups are more in favor of FHH education than those who are non-Hispanic white. Given that our findings suggest that participants who are members of diverse racial and ethnic groups are eager to learn more about FHH, culturally appropriate FHH education targeting members of diverse racial and ethnic groups should be designed, created, and offered in a culturally sensitive manner. 

Our study results also highlight the preferred topics of FHH education among college students. Notably, interpreting FHH results, using FHH to improve health, and FHH collection strategies are the three most highly desired topics among participants. These findings suggest that the participants in our sample with a desire to learn about FHH are eager to learn more about the application and use of FHH in health promotion and disease prevention. Participants also want to know why FHH is important for their health (as the fourth desired topic), and what FHH is (as the fifth desired topic). As such, future FHH educational efforts designed for college students should emphasize the proper use of FHH, the importance of FHH, and the FHH knowledge required to improve young adults’ skill and self-efficacy in FHH collection and application of the knowledge gained to disease prevention efforts. 

In addition to preferred topics in FHH education, this study provides insight into college students’ desired FHH educational strategies. Our results show that most participants report computer-based technology as their preferred learning method. This finding is not surprising, as college students are very familiar with using computers and feel comfortable with digital health-based interventions. Previous studies have demonstrated the effectiveness of numerous computer-based health education intervention programs (e.g., nutrition and alcohol use educational interventions) offered for college students [49,50]. However, according to a systematic review of the literature that summarized the characteristics and effectiveness of FHH interventions and education, among the limited number of FHH interventions and education in college settings, none of them adopted computer-based technologies [11]. It is strongly recommended that future FHH educational efforts in college settings use cutting edge computer-based technologies to increase student participation and engagement. It is also interesting to note that the majority of our participants choose more than one educational strategy, which indicates that college students are willing to learn about this topic via multiple methods. Thus, it is critical to incorporate diverse learning strategies, such as discussion, demonstration, simulated games, and traditional writing materials, into the FHH education. 

Several limitations in this study should be noted. First, as this was a voluntary survey, it is possible that the study sample was affected by selection bias. The college students who completed the survey might be more aware of their FHH and be more interested in FHH than the general population. Compared with demographic characteristics of the enrolled student population from the institution, our sample has more women and Asian or Pacific Islander participants, which indicates that women and Asian or Pacific Islander college students may be more interested in this topic than other sectors of the population. Second, this study only assessed FHH educational needs in two campuses of a large university in the southern U.S. Although our study includes participants who are diverse in age, gender, race, and ethnicity, the results of this study may not be generalizable to all college students. Third, we do not collect data describing participants’ major areas of study. Given that the majors might be a potential factor influencing college students’ knowledge of FHH and interest in FHH education, future studies should examine participants’ major as a modifying factor. However, instead of asking majors, we asked our participants if they have taken a genetic/genomic course before or had ever enrolled in a course containing genetics/genomics-related information, but these two factors are not significantly associated with participants’ interest in FHH education. Fourth, the educational strategies options we provided in the questionnaire are not mutually exclusive. Lastly, although we utilized multiple strategies (e.g., providing monetary incentives, sending out three reminder emails, and carefully choosing the timing of survey distributing to avoid holidays and final exams) to promote participation, the response rate of this web-based survey study was still low (5.08%). The possible reason might include that college students did not frequently check their university email accounts, ignored email sending from the university bulk email system, lacked interest in this type of study, or were busying working on other priorities. Future FHH research/interventions focused on this population need to adopt more strategies to increase the participation. 

Despite these limitations, to the best of our knowledge, this is the first-of-its-kind needs assessment study to evaluate college student interest in FHH education and their preferred FHH educational topics and strategies. This study opens an initial window to assess college students’ educational needs in FHH and contributes to the development and implementation of future FHH educational efforts in university settings. Our results show that fewer than half of college students are interested in FHH education as FHH education is not a priority in their lives. Given that college is an important time for young adults to know their FHH and take preventive actions based on their disease risks, future FHH educational efforts should be designed and tailored based on their groups of interests in this topic. In addition, computer-based technology needs to be incorporated into FHH education at college settings.

## 5. Conclusions

To the best of our knowledge, this is the first-of-its-kind study to assess college students’ educational need of FHH. Our findings indicate that more than half of the participants in our study, however, report no interest in receiving education about FHH mainly due to low prioritization. Among those who express interest in obtaining FHH education, the desired learning topics include the ability to interpret FHH information, the application of FHH in disease prevention, and FHH information collection strategies. In addition, our findings indicate that computer-based learning is the most preferred educational method. Females, older individuals, those who have FHH in first-degree relatives, and participants who are members of racial and ethnic groups show greater interests in receiving FHH education. Educational strategies to promote college students’ awareness, collection, and use of FHH are needed.

## Figures and Tables

**Table 1:** Demographic characteristics of college students (N = 2276).

Characteristics	N (%)
Age: Mean (SD ^1^)	21.0 (3.4)
Gender	
Male	772 (34.0)
Female	1498 (66.0)
Birthplace	
Born outside of the U.S.	480 (21.1)
Born in the U.S.	1796 (78.9)
Ethnicity	
Non-Hispanic white	1032 (45.5)
Other	1238 (54.5)
Race	
African American	91 (4.0%)
Alaska Native or American Indian	21 (0.9%)
White or Caucasian	1423 (62.7%)
Asian or Pacific Islander	496 (21.9%)
Multiple races	158 (7.0%)
Others	81 (3.6%)
Marital status	
Married/living as married	138 (6.1)
Others	2138 (93.9)
Religion	
Christian (including Catholic, Protestant, and all other Christian denominations)	1462 (64.3)
Unaffiliated/none	528 (23.2)
Other	285 (12.5)
Have taken a course in genetics or genomics in college	
No	1921 (84.4)
Yes	355 (15.6)
Have taken a course containing genetics/genomics-related information in college	
No	1508 (66.3)
Yes	768 (33.7)

^1^ SD: standard deviation.

**Table 2:** Multiple linear regression analysis for college students’ interests in receiving FHH education.

Variable	β	SE	t
(Constant)		0.269	6.83
Age (in years) **	0.021	0.008	2.61
Gender (male/female) ***	0.206	0.508	4.05
Birthplace (born outside of the U.S./born in the U.S.)	−0.114	0.065	−1.76
Race/ethnicity (non-Hispanic white/other) *	0.105	0.049	2.13
Marital status (married or living as married/others)	−0.059	0.109	−0.54
Have taken a course in genetics or genomics in college (no/yes)	0.030	0.078	0.39
Have taken a course containing genetics/genomics-related information in college (no/yes)	0.114	0.059	1.94
Fruit consumption	0.030	0.015	1.94
Red meat consumption	−0.003	0.004	−0.87
FHH of major diseases in first-degree relatives (no or not sure/yes) *	0.185	0.061	3.00

FHH: family health history. *** *p* < 0.001, ** *p* < 0.01, * *p* < 0.05.

## Data Availability

As a data-sharing strategy was not included in the original application for institutional review board review, study data are not publicly available.
